# Minimum infectious dose for chikungunya virus in *Aedes aegypti* and *Ae. albopictus* mosquitoes

**DOI:** 10.26633/RPSP.2017.65

**Published:** 2017-07-08

**Authors:** Jeremy P Ledermann, Erin M Borland, Ann M Powers

**Affiliations:** 1 Division of Vector-Borne Diseases Centers for Disease Control and Prevention Fort Collins, CO United States of America Division of Vector-Borne Diseases, Centers for Disease Control and Prevention, Fort Collins, CO, United States of America.

**Keywords:** Aedes, chikungunya virus, epidemiology, Americas, Aedes, virus chikungunya, epidemiología, Américas

## Abstract

Understanding the ability of the chikungunya virus (CHIKV) to be transmitted by Aedes vectors in the Americas is critical for assessing epidemiological risk. One element that must be considered is the minimum infectious dose of virus that can lead to transmission following the extrinsic incubation period. This study aimed to determine the minimum infection rate for the two Aedes species studied. The results revealed that doses as low as 3.9 log_10_ plaque-forming units per mL (pfu/mL) of an Asian genotype CHIKV strain can lead to transmission by Ae. albopictus, and doses of at least 5.3 log_10_ pfu/mL from the same strain are needed for transmission from Ae. aegypti. These low infecting doses suggest that infected individuals may be infectious for almost the entire period of their viremia, and therefore, to prevent further cases, measures should be taken to prevent them from getting bitten by mosquitoes during this period.

## RESUMEN

Comprender la capacidad del virus del chikungunya (CHIKV) de ser transmitido por los vectores del género Aedes en la Región de las Américas es fundamental para evaluar el riesgo epidemiológico. Un elemento que debe tenerse en cuenta es la dosis infecciosa mínima de virus que posibilita la transmisión después del período de incubación extrínseco. El objetivo de este estudio ha sido determinar la tasa de infección mínima para las dos especies del género Aedes estudiadas. Los resultados indican que bastan dosis de tan solo 3,9 log10 unidades formadoras de placas por mililitro (ufp/ml) de una cepa de CHIKV del genotipo asiático para que se produzca la transmisión por Ae. albopictus, en tanto que para la transmisión por Ae. aegypti se necesitan dosis de al menos 5,3 log10 ufp/ml de la misma cepa. Estas dosis bajas indican que las personas infectadas podrían conservar el potencial infeccioso prácticamente durante todo el período de viremia y, por consiguiente, a fin de prevenir más casos, habría que tomar medidas para impedir que reciban picaduras de mosquitos durante ese período.

Chikungunya virus (CHIKV) is a positive-sense single-stranded RNA alphavirus belonging to the family *Togaviridae* that has been the focus of a global epidemic beginning in 2004 in coastal Kenya with spread to the Americas in 2013. The virus is responsible for more than 3 million cases of debilitating arthralgia in many countries in the tropics and subtropics over the past decade. Interestingly, there have been multiple genotypes of CHIKV involved in the global spread of the virus during this time. In Kenya, in 2004, a strain of the East/Central/South African (ECSA) genotype caused two outbreaks in the eastern coastal areas of the country. This genotype was not unexpected given the geographic location of the outbreak. This lineage then expanded its range, moving into multiple islands of the Indian Ocean before arriving in India, where, for the first time (2006), the ECSA genotype was transmitted outside of Africa. The ECSA Indian Ocean lineage continued to move throughout Southeast Asia. The Asian genotype virus was also found to be circulating at low levels in Asia, but this transmission was scarcely noticed, given the scope of the ECSA genotype spread. However, the Asian genotype moved to novel areas of the Western Pacific (New Caledonia and the Federated States of Micronesia) before subsequently being detected in the Caribbean late in 2013 (1).

Throughout the epidemic, the virus was transmitted by its urban vectors (subgenus *Aedes (Stegomyia)*). From the earliest urban outbreaks, the virus was primarily transmitted by *Ae. aegypti* mosquitoes, but recent activity has also been confirmed to be associated with *Ae. albopictus*, as CHIKV outbreaks have been documented in places with no presence of the historical primary vector, such as Italy (2). In addition, other *Ae. (Stg.)* vectors (e.g., *Ae. hensilli*) have sustained outbreaks in areas where these species are prominent.

In anticipation of the virus reaching the Americas, there was considerable concern about the ability of local populations of mosquitoes to vector the introduced virus. Because the only *Ae. (Stg.)* species known to be in the Americas are *Ae. aegypti* and *Ae. albopictus,* a number of studies were undertaken to assess the vector competence of both species (3–7). Typically, both species were found to be readily infected by and competent vectors for either an ECSA strain or the introduced Asian genotype strain. However, as with most artificial infection studies, a high dose of virus (∼6 log_10_ plaque-forming units per mL (pfu/mL)) was usually used to infect the mosquitoes. The goal was to determine the minimum infection rate for each species. This information would allow for estimation of the length of time a viremic individual would be infectious. Because CHIKV-infected individuals have been estimated to have detectable levels of viremia for at least seven days (8), understanding the smallest dose needed to infect humans would provide significant public health preparedness information.

## MATERIALS AND METHODS

### Virus isolates

The viruses used for laboratory mosquito infections were obtained from the Arbovirus Reference Collection at the U.S. Centers for Disease Control and Prevention (CDC) (Fort Collins, Colorado, United States). CHIKV strain BVI 99660 was obtained from human serum samples from locally transmitted cases in the British Virgin Islands in 2014.

### Laboratory mosquito infections

Three- to four-day-old adult *Ae. aegypti* collected in Puerto Rico in 2015 (“PR”) and *Ae. albopictus* collected in Lake Charles, Louisiana, United States, in 1990 (“LC”) were fed on blood meals containing CHIKV strain BVI 99660 at various dilutions. The blood meals contained equal parts of virus, fetal bovine serum (FBS) with 10% sucrose, and sheep blood washed with phosphate-buffered saline and packed by centrifugation. A Hemotek feeding system (Discovery Workshops, Accrington, United Kingdom) was used to deliver the blood meal to the mosquitoes for 1 hour at 37ºC. The fully engorged females were collected and placed into a humidified environmental chamber and held at 28ºC for 8 or 12 days until processing. The input virus titer for the blood meal titer was determined using a plaque assay.

After the intrinsic incubation period, the mosquitoes were cold-anesthetized and decapitated and each head and body placed into separate 1.7 mL tubes. A 450 μL aliquot comprised of 400 μL of Dulbecco’s modified Eagle’s medium (DMEM) supplemented with 10% FBS, 100 U/mL of penicillin and streptomycin, and 1 U/mL of fungizone and gentamycin was added to each tube, and the sample was homogenized using a micropestle. The supernatant was clarified by filtration through a 0.2 μM syringe filter and stored at –70ºC until use (9). Filtrates were tested for cytopathic effect (CPE) on Vero cells held at 37°C with 5% CO_2_ for five days. A CPE-positive mosquito abdomen indicated a midgut infection whereas a CPE-positive mosquito head indicated a disseminated viral infection. The percent infected (proportion infected in a cohort, or prevalence) was calculated as the number of CPE-positive bodies out of the total number of bodies processed. The percent disseminated (proportion of samples in which the virus had been disseminated) was calculated as the number of CPE-positive heads out of the total number of CPE-positive bodies.

Saliva was collected before dissection from 20 mosquitoes per experiment. Mosquitoes were allowed to salivate into a glass capillary tube charged with 5 μL of Cargille type B immersion oil (Cargille Laboratories, Cedar Grove, New Jersey, United States) for 1 hour (10). The oil-filled end of the tube was broken off into the 1.7 mL tube containing the 450 μL of the DMEM solution, and the tubes were centrifuged at 4 500 x *g* for 5 minutes. Saliva samples were stored at –80°C until RNA extraction was performed.

### Viral nucleic acid extraction and detection

Viral RNA was isolated using the QIAamp Viral RNA protocol (QIAGEN Inc., Valencia, California, United States). Total RNA was extracted from 100 μL of mosquito homogenate or from the saliva and eluted from the kit columns using 60 μL of elution buffer. The RNA was stored at –70ºC until use.

Real-time quantitative reverse transcription polymerase chain reaction (qRT-PCR) was used to detect and quantify CHIKV nucleic acid from mosquito saliva. The previously described chikungunya virus–specific primer and probe sets were used (11). The qRT-PCR assays were performed using the QuantiTect Probe RT-PCR reagent kit (QIAGEN Inc.). Briefly, a 50 μl total reaction volume consisted of kit components, 10 μL of RNA, 400 nanomolar (nM) of each primer, and 150 nM of probe. The reactions were subjected to 45 cycles of amplification in an iQ5 Real-Time PCR Detection System (Bio-Rad, Hercules, California) following the manufacturer’s protocol. The detection limit for CHIKV was found using the previously described techniques (12) and was cycle threshold (Ct) 38.0, which is equivalent to approximately 1.0 pfu/mL. In addition, each qRT-PCR run included standards to generate a quantitative RNA curve. The standard curve was completed by serially diluting the virus stock and extracting the RNA from each dilution, according to the previously mentioned RNA extraction protocol, while simultaneously titrating each dilution in a standard plaque assay. A curve correlation coefficient of ≥ 0.950 and a 90%–100% PCR efficiency was used to validate each detection assay.

## RESULTS

Infection of both *Ae. aegypti* (PR) and *Ae. albopictus* (LC) occurred with any dose > 3 log_10_ pfu/mL of the Asian genotype virus input. The minimum dose infecting *Ae. aegypti* and *Ae. albopictus* where virus could be detected in the saliva of at least one mosquito is between 4.9–5.3 log_10_ pfu/mL and 2.9–3.9 log_10_ pfu/mL respectively (Table 1). The maximum percent infected (based on CPE analysis) for *Ae. aegypti* mosquitoes was 95.8% (with a dose of 6.3 log_10_ pfu/ml); at the lowest dose demonstrating transmission (5.3 log_10_ pfu/mL) the percent infected was 60.0%. A lower virus exposure dose was observed for *Ae. albopictus* mosquitoes, with a dose as low as 3.9 log_10_ pfu/mL of CHIKV in that species resulting in a 35.0% infection rate, and the maximum dose evaluated (5.7 log_10_ pfu/mL) resulting in an 84.6% infection rate. In both species, when infection occurred, virtually all mosquitoes developed a disseminated infection. This was detected even when infection rates were as low as 13.9%. In all cases, for both mosquito species, the lowest dissemination rate detected was 75.0% (in *Ae. aegypti*, with a blood meal titer of 4.9 log_10_ pfu/mL).

**TABLE 1.** Percent infection of bodies and heads of two strains of *Aedes* mosquito (based on cytopathic effect (CPE) analysis) and virus quantitation in saliva after exposure to CHIKV strain BVI 99660,^a^,^b^ Fort Collins, Colorado, United States, 2016

Mosquito strain^c^	Number tested	Infecting dose (log_10_ pfu^d^/mL)	CPE analysis		Virus quantitation in saliva	
			Body (%)	Head (%)	Pos^e^ (%)	Titer (pfu)
*Ae. aegypti* (PR)	36	4.6	13.9	80.0	0	0
	28	4.9	14.3	75.0	0	0
	10	5.3	60.0	100.0	33.3	505
	37	5.6	73.3	100.0	53.3	935
	36	6.1	91.7	100.0	75.0	5 000
	24	6.3	95.8	100.0	36.8	3 545
*Ae. albopictus* (LC)	30	2.9	0	0	0	0
	40	3.9	35.0	92.9	66.7	1 921
	32	4.6	71.9	95.7	66.7	2 334
	39	5.7	84.6	97.0	94.4	39 100

a Chikungunya virus (human serum sample collected in the British Virgin Islands in 2014).

b All results are for Day 8 post-exposure except the 6.3 log_10_ dose, which was for Day 12 post-exposure.

c *Ae. aegypti* collected in Puerto Rico in 2015 (“PR”) and *Ae. albopictus* collected in Lake Charles, Louisiana, United States, in 1990 (“LC”) (3- to 4-day-old adults).

d Plaque-forming units.

e “Positive” (proportion of CPE-positive heads with saliva positive for the virus based on quantitative reverse transcription polymerase chain reaction (qRT-PCR)).

Dissemination occurred with nearly all infections in both species, and the percentage of mosquitoes transmitting the virus increased with higher virus titers in the blood meal. In *Ae. aegypti*, when no transmission was detected, the proportion infected was less than 15%. Transmission in *Ae. albopictus* occurred at a lower dose than *Ae. aegypti* (3.9 log_10_ pfu/mL versus 5.3 log_10_ pfu/mL respectively). However, transmission rates reached at least 75% in both species. The maximum titer of CHIKV in saliva was 5 000 pfu after exposure to 6.1 log_10_ pfu/mL of virus for *Ae. aegypti* and 39 100 pfu after exposure to 5.7 log_10_ pfu/mL for *Ae. albopictus* mosquitoes. However, variation of the viral titer in the saliva was not proportional to the input dose; all saliva samples contained a 5x10^2^–5x10^3^ viral titer. Variation in the saliva virus titer was only found at the highest dose in *Ae. albopictus*, where a titer of 4x10^4^ was found.

## DISCUSSION

Understanding the ability of a mosquito species to be infected by and transmit a virus is a critical element in gauging the epidemiological risk of local populations. With the introduction of CHIKV in the Americas with a strain of virus from, unexpectedly, the Asian genotype, it was critical to assess how populations of both known vectors, *Ae. aegypti* and *Ae. albopictus*, would be able to transmit CHIKV. Historically, various populations of both species have been assessed for infection with CHIKV of various lineages (13, 14). These early studies indicated that both vector species would serve as competent vectors, but *Ae. albopictus* was often found to be more competent in laboratory studies. This was an interesting and unexpected finding at the time, because (until 2006) no CHIKV outbreak had ever been documented as associated with *Ae. Albopictus*.

In preparing for the entry of CHIKV into the Americas, a number of studies sought to evaluate American populations of *Ae. aegypti* and *Ae. albopictus* for infectivity with the anticipated lineage of CHIKV derived from the Indian Ocean lineage of the ECSA genotype. One study examined *Ae. albopictus* from the Eastern United States using a range of blood meals (5.3x10^3^–1.5x10^5^ pfu/mL) and determined that the percent infected ranged from 31% to 86% (6). The study also examined transmission rates, which never exceeded 50%. A similar single-species study focused on *Ae. aegypti* from the French West Indies and French Guiana (where the virus was first established in the Americas) (4). The study used local populations of *Ae. aegypti* with only high titers (6.0 or 7.5 log_10_ pfu/mL) of the infecting virus (the ECSA genotype). Infection rates were 38%–62% for the 6 log dose and 89%–100% for the higher dose. Another study looked at infection in both *Ae. albopictus* and *Ae. aegypti* from the U.S. state of Florida (F1) and other (long- established) colonies (including the LC strain used in this study) using blood meals with titers of approximately 6 log_10_ pfu/mL (5). All mosquito species and virus strains tested had infection rates greater than 90% at seven days post-exposure. Interestingly, the study also examined the timing of the dissemination of the virus within the mosquitoes and found that both strains of *Ae. albopictus* (Florida and LC) disseminated equally well and more efficiently than either *Ae. aegypti* strain*.* However, no transmission rates were determined in the study, and only high infecting virus doses were used. In addition, all of these studies used ECSA viral lineage strains, which may have resulted in properties very different than those in mosquitoes infected with an Asian genotype lineage.

One very comprehensive study examined 35 populations of *Ae. albopictus* and *Ae. aegypti* from 10 countries in the Americas (ranging geographically from Argentina to the United States) to determine dissemination and transmission rates using both ECSA strains and an Asian genotype strain (7). Overall, high dissemination rates were found in all *Ae. albopictus* and *Ae. aegypti* populations tested; only four had dissemination rates less than 83% at Day 7 post-exposure. All *Ae. albopictus* and *Ae. aegypti* populations exposed to the Asian genotype strain had infection rates of at least 90% but the infecting blood meal dose was extremely high (7.5 log_10_ pfu/mL). This was interesting given that no infection assessment was performed directly but the high dissemination rates indirectly suggest the infection rate was equally high. The transmission rates were found to vary significantly with the population examined. Overall, *Ae. albopictus* infected with the Asian genotype strain had lower transmission rates than corresponding populations of *Ae. aegypti.* The study also included a transmission efficiency element that suggested the possibility of the presence of a salivary gland escape barrier, but the very high infecting dose may have biased overall infection and dissemination values.

In this study, the goal was to assess U.S. mosquito populations of *Ae. albopictus* and *Ae. aegypti* using a relevant strain of virus (the Asian genotype) at a range of doses to determine the minimum infectious dose. Like the earlier studies, high rates of dissemination were found, virtually independent of blood meal virus titer or percentage of mosquitoes infected. Basically, if the mosquitoes became infected, the virus spread throughout the mosquito population, suggesting no salivary gland infection barriers exist in either *Ae. albopictus* or *Ae. aegypti.* In contrast, infection rates were linked to the blood meal virus titer, indicating that a midgut infection barrier is present, most likely in both species but more pronounced in *Ae. aegypti*. Transmission rates in the two species also increased with higher blood meal virus titers, which was somewhat unexpected given that the dissemination rates in both species, which were high, did not correlate with infecting dose. The variation in the transmission rates suggests the presence of a salivary gland escape barrier, as has been previously speculated.

This study showed that infecting titers at least as low as 3.9 log_10_ pfu/mL (in *Ae. albopictus*) and 5.3 log_10_ pfu/mL (in *Ae. aegypti*) could lead to transmission, indicating the infectious period of a viremic individual is proportionally longer than expected. Epidemiology studies have shown that viremia in infected humans can be detected at up to 10 days post–illness onset, with titers of at least 103.9 pfu/mL at every time point tested (8). Those findings, combined with the results of this study, suggest that the vast majority of individuals could infect mosquitoes during most of their viremic period (which typically lasts for approximately one week). This may be a major epidemiological factor in the high attack rates found in areas where the virus is newly introduced.

### Limitations

This study had some limitations, such as not using a range of different mosquito populations, and the use of long-colonized strains (although previous work has shown the latter trait is not a contributing factor in infection and dissemination rates in *Ae. albopictus* LC).

### Conclusions

This study has shown that acutely ill individuals are highly infectious to mosquitoes for extended periods of time. Therefore, in addition to conventional measures to reduce the risk of mosquito bites population-wide, such as larval source reduction and use of larvicidal products, the use of additional measures to prevent infected individuals from getting bitten by mosquitoes, which could lead to further infections, are necessary for public health protection during active transmission events. Additional measures could include the use of mosquito repellents; long sleeves and/or pants; and bed nets (particularly during daytime hours) by already-infected individuals. Adding these additional measures to interventions may help control clusters of infections and reduce the scope of the overall outbreak.

## Acknowledgments.

The authors thank Roberto Barrera and John-Paul Mutebi of the U.S. Centers for Disease Control and Prevention (CDC) for providing the mosquitoes used in this study.

## Disclaimer.

Authors hold sole responsibility for the views expressed in the manuscript, which may not necessarily reflect the opinion or policy of the CDC, the *RPSP*/*PAJPH*, or the Pan American Health Organization *(*PAHO).
